# Recovery kinetics of dual AAV-mediated human otoferlin expression

**DOI:** 10.3389/fnmol.2024.1376128

**Published:** 2024-06-17

**Authors:** Jonathan B. Sellon, Kathy S. So, Andrew D'Arcangelo, Sarah Cancelarich, Meghan C. Drummond, Peter G. Slade, Ning Pan, Tyler M. Gibson, Tian Yang, Joseph C. Burns, Adam T. Palermo, Lars Becker

**Affiliations:** ^1^Decibel Therapeutics, Inc., Boston, MA, United States; ^2^Regeneron Pharmaceuticals, Inc., Tarrytown, NY, United States

**Keywords:** DFNB9, otoferlin, OTOF, *ex vivo*, explant, dual hybrid, gene therapy, vestibular

## Abstract

Deafness-causing deficiencies in *otoferlin* (*OTOF*) have been addressed preclinically using dual adeno-associated virus (AAV)-based approaches. However, timing of transduction, recombination of mRNA, and protein expression with dual hybrid AAV methods methods have not previously been characterized. Here, we have established an *ex vivo* assay to determine the kinetics of dual-AAV mediated expression of *OTOF* in hair cells of the mouse utricle. We utilized two different recombinant vectors that comprise DB-OTO, one containing the 5′ portion of *OTOF* under the control of the hair cell-specific *Myo15* promoter, and the other the 3′ portion of *OTOF*. We explored specificity of the *Myo15* promoter in hair cells of the mouse utricle, established dose response characteristics of DB-OTO *ex vivo* in an OTOF-deficient mouse model, and demonstrated tolerability of AAV1 in utricular hair cells. Furthermore, we established deviations from a one-to-one ratio of 5′ to 3′ vectors with little impact on recombined *OTOF*. Finally, we established a plateau in quantity of recombined *OTOF* mRNA and protein expression by 14 to 21 days *ex vivo* with comparable recovery timing to that *in vivo* model. These findings demonstrate the utility of an *ex vivo* model system for exploring expression kinetics and establish *in vivo* and *ex vivo* recovery timing of dual AAV-mediated *OTOF* expression.

## Introduction

The majority of cases of nonsyndromic profound congenital deafness are genetic with an autosomal recessive pattern of inheritance (DFNB) (Duman and Tekin, 2012). To address hearing loss in patients with genetic causes of deafness, adeno-associated virus (AAV) gene therapy provides a promising alternative to cochlear implants given the potential for improving perception of music and speech in noisy environments by eliminating limitations on frequency selectivity due to current spread. AAV is particularly attractive since it has been demonstrated to efficiently transduce many dividing and non-dividing cell types and is less immunogenic than other viral vectors (Kay, 2011). To date, there have been a variety of studies demonstrating the promise of AAV gene therapy to restore hearing function, including therapies for mutations of *STRC* (Shubina-Oleinik et al., 2021), *VGLUT3* (Akil et al., 2012), *TMC1* (Askew et al., 2015), *KCNQ1* (Chang et al., 2015), and *CLRN1* (Dulon et al., 2018). We focused on DFNB9 auditory neuropathy (MIM 601071), which results from mutations of *OTOF* which encodes otoferlin, a calcium sensor for synaptic exocytosis in inner hair cells of the inner ear. Otoferlin-mediated neurotransmitter release is essential for proper communication with the auditory nerve (Roux et al., 2006) and is also involved in replenishing IHC synaptic vesicles (Pangršič et al., 2010; Wong et al., 2014). Patients with OTOF deficiency due to biallelic *OTOF* mutations have dysfunctional IHC synapses with the auditory nerve, leading to deafness (Rodríguez-Ballesteros et al., 2008). Recently, promising work demonstrated recovery of hearing in a mouse model of *otoferlin* deficiency using AAV-based gene therapy (Akil et al., 2019; Al-Moyed et al., 2019; Qi et al., 2023; Zhang et al., 2023), suggesting the potential of gene therapy in DFNB9 patients.

While promising for functional recovery, AAV has packaging capacity of less than 5 kb (Reisinger, 2020), which limits the utility of single vectors for treating monogenetic disorders of hearing, many of which are larger than 5 kb (Jones and Jones, 2014) including *otoferlin*. Because the coding sequence for the complete *OTOF* cDNA exceeds the packaging capacity of an AAV1 vector, DB-OTO (AAV1-Myo15-hOTOFv5) is composed of two AAV1 vectors which encode the 5′ and 3′ components, of human *OTOF* (Ghosh et al., 2011). When present in the same cell, the two vectors reconstitute a functional *OTOF* gene cassette for expressing full-length OTOF protein isoform 5. While this dual vector technique has been shown to restore hearing in OTOF-deficient mice (Akil et al., 2019; Al-Moyed et al., 2019; Qi et al., 2023; Zhang et al., 2023), little is known about the dynamics of dual vector recombination. An understanding of these kinetics will enable a better understanding for the timing of recovery of hearing in human patients treated with dual vector AAV-based gene therapy and for the kinetics of dual vector recombination therapy in general.

In addition to packaging constraints, promoter identification is another important aspect of vector selection for an AAV-based gene therapy. To avoid potential off-target toxicity, we utilize a hair cell specific *Myo15* promoter on the 5′ vector end to restrict expression to hair cells. While use of this promoter may provide therapeutic benefit, it adds complication for answering questions regarding dual vector recombination due to lack of *Myo15* expression in publicly available cell lines. Additionally, while a dual vector recombination strategy recovers hearing thresholds in mouse studies (Akil et al., 2019; Al-Moyed et al., 2019; Qi et al., 2023; Zhang et al., 2023), potential variability due to surgical delivery to the inner ear, make determining recombined RNA and protein kinetics challenging for *in vivo* dosed samples.

To circumvent these challenges for determining dual vector recombination kinetics, we utilized mouse utricular explant culture to demonstrate the specificity of the *Myo15* promoter in vestibular hair cells and assess expression kinetics of recombined *OTOF*. Previous work has established the mouse utricle as being a robust system for hair cell culture, with survival times of up to 28 days (Burns et al., 2012). Additionally, recent work has demonstrated that dual-AAV vectors can transduce the vestibular end organs of mice with efficiency and minimal damage to hair cells using the AAV-ie serotype (Tan et al., 2019; Chen et al., 2022). Thus, to explore kinetics of dual-AAV mediated transgene expression we first explore split-GFP expression driven by the cell-specific *Myo15* promoter in the mouse utricle. By utilizing a split-GFP transgene we can establish both tolerability of the AAV1 serotype and efficiency of dual vector recombination in the adult mouse utricle. After establishing specificity of delivery to utricular hair cells with split-GFP vectors we explore *OTOF* expression via dual-AAV delivery using utricular tissue from utricles of adult *Otof^Q828X^* mice, which we developed to be transgenic for an *OTOF* nonsense mutation. This mutation models a prevalent human mutation that leads to expression of a non-functional OTOF protein and congenital deafness (Migliosi, 2002). We establish methods to reliably evaluate transcript levels of *OTOF* in single utricles by reverse transcription quantitative polymerase chain reaction (RT-qPCR), as well as protein content by immunohistochemistry (IHC). After establishing these methods, we determine the length of time to detect full length recombined *OTOF* transcripts, the timing for transcripts to plateau, the timing for OTOF protein to plateau, and established deviations from a one-to-one ratio of 5′ to 3′ vectors with little impact on recombined *OTOF*. Finally, we compare recovery timing in this *ex vivo* system to *in vivo* delivery of DB-OTO to *Otof^Q828X/Q828X^* mice to assess translatability of the *ex vivo* model. These findings demonstrate the utility of an *ex vivo* system for exploring dual-AAV mediated expression kinetics.

## Materials and methods

### *Otof^Q828X^* mice generation

CRISPR-Cas9-mediated knock-in was utilized to make a point mutation in the OTOF amino acid sequence at position 828 in FVB mouse zygotes. We selected this truncation mutation because the orthologous human mutation at position 829 of OTOF is well characterized as pathogenic allele (Rodríguez-Ballesteros et al., 2008). Single guide RNAs (sgRNAs) with low off-target cutting potential were screened in cell lines via the Surveyor Cel-1 Mutation Detection assay (Transgenomic, Gaithersburg, MD, United States) (Qiu et al., 2004). An sgRNA targeting sequence with the protospacer motif beginning 6 nucleotides upstream of the intended C > T point mutation was identified to have the highest cutting efficiency at 29.7%.

Th sgRNA with the highest cutting efficiency was assembled into a ribonucleoprotein complex with Cas9 endonuclease. Together, with a donor DNA oligonucleotide, the complex was microinjected into FVB/NJ mouse zygotes. The donor DNA oligonucleotide was a 143-nucleotide sequence containing both left and right homology arms flanking the intended C > T point mutation that changes the glutamine at the 828 position of Otof to a premature termination.

Zygotes that were microinjected were embryo transferred into pseudo-pregnant FVB/NJ females. Genomic PCR and Sanger sequencing were utilized to determine the presence of the desired mutation in viable progeny. Founder mice were selected and bred with FVB/NJ wt mice to generate heterozygous F1 progeny and these F1 progeny were used to establish further generations to expand a colony of mice homozygous for the Q828X mutation. Presence of the mutation was established via PCR-mediated genotyping and DNA sequencing.

### Animal housing and care

*Otof-Q828X* homozygous mice were housed in the Decibel Therapeutics, Inc. animal care facility. Mutant mice were housed in groups of up to 5 under controlled conditions of 64°F to 79°F and 30 to 70% relative humidity with 12-h cycles of light and dark. All experiments were conducted in accordance with the Institutional Animal Care and Use Committee (IACUC) protocol 2020–011.

### *Ex vivo* explant culture

All animal use (protocol 2018-008) were approved by the Animal Care and Use Committee of Decibel. 6–8 weeks old of males of C57BL/6 J (The Jackson Laboratory, 000664), and *Otof^Q828X/Q828X^*, and FVB/NJ (The Jackson Laboratory, 001800) were used. After animals were sacrificed by CO2 euthanasia, utricles were dissected in ice-cold DMEM/F-12 solution (11,039,021, Gibco) and cultured in DMEM/F-12, GlutaMax culture media (10,565,018, Gibco) supplemented with 10% FBS (F4135, Sigma) and 2.5 ug/ml Ciprofloxacin (AC456880050, Fisher Scientific) in glass bottom culture dishes (10810-054, Matsunami Glass). When AAV was added to the media, 250 μL of the culture media was used and AAVs were left in the culture media for 3 days before washing out. This timing was chosen for flexibility and enabled uniform culture timing across all studies. Additionally, we expect similar expression for shorter times in virus based on other AAV cell-surface-binding kinetics reports demonstrating rapid uptake on the order of hours (Asokan et al., 2008). Utricles were cultured with 2 mL fresh media for the duration based on the study design. The culture media was changed every 3–4 days.

### Immunohistochemistry

At the end of the culture, samples were fixed with fresh 4% PFA in 1X PBS for 1 h at room temperature (RT) and rinsed with 1X PBS for 3 times with 5 min per wash. Tissues were blocked with 10% normal goat serum, 0.5% TritonX-100, in PBS at pH 7.4 for 1 h at room temperature, followed by incubation with primary antibodies diluted 0.5% TritonX-100 in 1X PBS overnight at 4°C. The next day, after washing with PBS, tissues were incubated with secondary antibodies diluted in blocking solution (2% serum) for 3 h at room temperature. After PBS washing, tissues were mounted in Slowfade Diamond Antifade Mounting Media (DAKO) (ThermoFisher Molecular probes, s36963). Antibodies against the following markers were used: anti-OTOF (Ms IgG1, Abcam 53,233, 1:200), anti-POU4F3 (1:200, Santa Cruz Biotechnology, sc-81980), Sox2 (1:500; Abcam, ab97959), Secondary antibodies were conjugated with Alexa Fluor 488, 568 and 647 (Invitrogen A-21240, A-11036, A-21447. A10037, A-21206).

When the anti-POU4F3 (Santa Cruz Biotechnology sc-81980) and anti-OTOF antibodies were used in the same experiment, the anti-POU4F3 antibody was pre-conjugated with the CF568 fluorophore (Biotium, #92235). Following the standard IHC protocol, utricles were labeled with fab goat anti-mouse Alexa-647 antibody (Jackson Immunoresearch, 115-607-185) against the Ms. IgG1 Otof primary antibody. After washing the secondary antibody 3 times with 1X PBS, the pre-conjugated Pou4f3 antibodies were applied to the samples in 0.5% TritonX-100 in 1X PBS and incubated at 4°C overnight. The following day, the samples were washed 3 times with 1X PBS before mounting.

### Image quantification

In Imaris 9.8.1 we created spots around hair cells using signal from the Pou4f3 channel and count the number of hair cells. The radii of these spots were enlarged to better capture the OTOF signal outside the cell nucleus using a custom python script with parameters selected to capture the largest number of hair cell nuclei and cytosolic protein quantity (Surface Grain Size = 0.750 μm, Diameter Of Largest Sphere = 5.00 μm, Manual Threshold Value = 1039.96, Manual Threshold Value B = 3920.06, Region Growing Estimated Diameter = 3.50 μm, Filter Seed Points Quality above 10.7, and filter surfaces voxels above 10.0). Mean intensity of OTOF signal in Q828X negative control images were calculated and was used as threshold to classify OTOF-positive signal in AAV treated groups. Hair cells that had OTOF signal above the threshold were counted as OTOF positive hair cells. Thresholds for determining OTOF positive cells were determined for each study separately. All images within the same study figure were captured with the same laser power, gain and bit depth to allow for comparison within a study.

### RNA extraction and quantitative RT-PCR for *ex vivo* cultures

RNA from utricles isolated using Arcturus PicoPure RNA Isolation Kit (Applied Biosystems, KIT0204) following manufacturer’s instructions for cell pellets/Macro LCM caps, with an additional DN ase step as described subsequently. After culture, utricles were lysed individually in 100 μL Picopure RNA lysis buffer, incubated at 42 degrees C for 30 min, and the samples were stored at −80°C until further processing. DNase digestion was additionally performed after Picopure wash buffer I was conducted with 10 μL DNase with 30 μL RDD buffer (RNase-Free DNase Set, Qiagen, 79,254) for each sample. After purification, RNA was eluted in 24 μL PicoPure elution buffer. All RNA was used for reverse transcription with the Superscript IV First Strand cDNA synthesis kit (ThermoFisher Scientific, 18,091,050), following the manufacturer’s instructions. Half of the RNA was used for with reverse transcriptase and the other half without reverse transcriptase with random hexamers.

RT-qPCR was performed with Taqman Fast Advanced Master Mix (ThermoFisher Scientific, 4,444,556) using the QuantStudio 6 Pro Real-Time PCR System and software (ThermoFisher Scientific). The following assays were used: *hOTOF10* for human otoferlin (IDT, Custom primers/probe design: Fwd Primer: 5’CGCCTCAAGTCCTGCAT, Rev. Primer: 5’ACAGCCTCAGCTTGTCC, Probe: 5′−/6-FAM/GCAGCAGGC/ZEN/CAGGATGCTGC/31AbkFQ/ -3′, *Cxcl14* (ThermoFisher, Mm00444699_m1), *Eif1* (Thermofisher, Mm00783932_s1), *Lmo1* (ThermoFisher, Mm01168131_m1), *Sox2* (ThermoFisher, Mm03053810_s1). ΔC_T_ values were obtained from two technical replicates per cDNA sample. Relative quantity was calculated from the mean and 95% confidence interval, 2^–∆∆Ct^, where ΔC_T_ = C_T_ (a target gene: OTOF) – C_T_ (reference gene) and ΔΔC_T_ = ΔC_T_ (test samples) – average [ΔC_T_ (untreated)].

### *In vivo* AAV delivery and sample collection

Male and female *Otof^Q828X/Q828X^* mice 7–13 weeks old were administered DB-OTO into the scala tympani space of the left cochlea via the round window membrane (RWM). DB-OTO was formulated in Dulbecco’s phosphate buffered saline (Mg + and Ca+) with 0.001% w/v poloxamer 188. Titer of the DB-OTO dosing solution was 1.0e14 vg/mL and 5 animals per timepoint group were dosed with an approximately 1:1 vector genome ratio of the 5′ and 3′ vectors in a total of 2 μL (2.0e11 vg/ear). Briefly, the AAVs were delivered by making a postauricular incision to expose the bulla, and then the bulla periosteum was gently separated to allow access to the RWM. After creating a hole in the bulla, the RWM was punctured, and DB-OTO was delivered using a machine-pulled (Sutter, P-30) borosilicate glass capillary (Drummond, 2-000-010) connected via a tubing (Microlumen, 039-0.5; SCI, BB31695-PE/5) to a 2 μL syringe (Hamilton, 7,002) and the whole volume was slowly injected into the Cochlea.

Animals were sacrificed using CO2 following all IACUC protocol and guidelines. Temporal bones were dissected and placed in ice-cold DMEM/F12 media. All excess muscle tissue and blood vessels were trimmed away, and the vestibular system was separated from the cochlea. An opening in the apex of the cochlea was created using a scalpel to allow for better lysis buffer access. The dissected cochlea was placed in a cryotube, flash frozen in liquid nitrogen, and stored at –80°C.

### RNA extraction and quantitative RT-PCR for *in vivo* samples

Total RNA from mouse cochlea was extracted using the MagMAX-96 for Microarrays Total RNA Isolation Kit (ThermoFisher, AM1839). 750 μL TRI Reagent was used to homogenize each cochlea and 150 μL chloroform was added to the homogenate. RNA purification and DNase digestion were then performed according to the manufacturer’s No spin Procedure. After the RNA elution step, RNase inhibitor was added to a final dilution of 1:40 to prevent RNA degradation. The RNA was quantified with the Qubit RNA High Sensitivity Assay (ThermoFisher, Q32852), and the quality of select samples was assessed with the Agilent Bioanalyzer RNA Pico kit (Agilent, 5,067-1,513). RNA samples were stored at −80°C.

100 ng RNA was reverse transcribed with random hexamers using the manufacturer’s protocol for the Superscript IV First Strand Synthesis System. Recombined *OTOF* expression was evaluated using custom designed primers as described above. Each sample was analyzed using 4 technical replicate RT-qPCRs with standard Taqman Fast Advanced Mastermix thermocycling conditions. A dilution series from 4.15E9 to 4.15 copies of hOTOF synthetic template was included for absolute quantification.

### Plasmid DNA generation

AAV transfer plasmids encoding human OTOF isoform under regulation of the *smCBA* promoter were generously donated by William Hauswirth and was used as a precursor to generate the plasmids used in this study.

All other plasmids were generated using standard gene synthesis and plasmid subcloning methods (Genscript, United States).

### Adeno-associated virus production and titration

All AAVs used in this study were generated by Packgene (Worcester, MA) using standard triple-transfection production in HEK293 cells with iodixanol gradient purification. AAV preps underwent independent tittering to confirm the concentration of genome-containing capsids, using standard protocols employing droplet digital PCR (ddPCR) methods.

Briefly, AAV preps were treated with DNAse I to remove non-encapsidated DNA, and vector genomes were released from the capsid using proteinase K. Liberated vector genomes were mixed with ddPCR master mix, PCR primers, and PCR probes and reactions were run using the QX200 AutoDG ddPCR system (Bio-Rad). The 5′ vector was run against Bio-Rad Assay ID #dCNS439296206, while the 3′ vector was run against Bio-Rad Assay ID #dCNS284272407.

## Results

### The *Myo15* promoter restricts expression of GFP to utricular hair cells and can be driven by dual vectors

**Figure 1:**
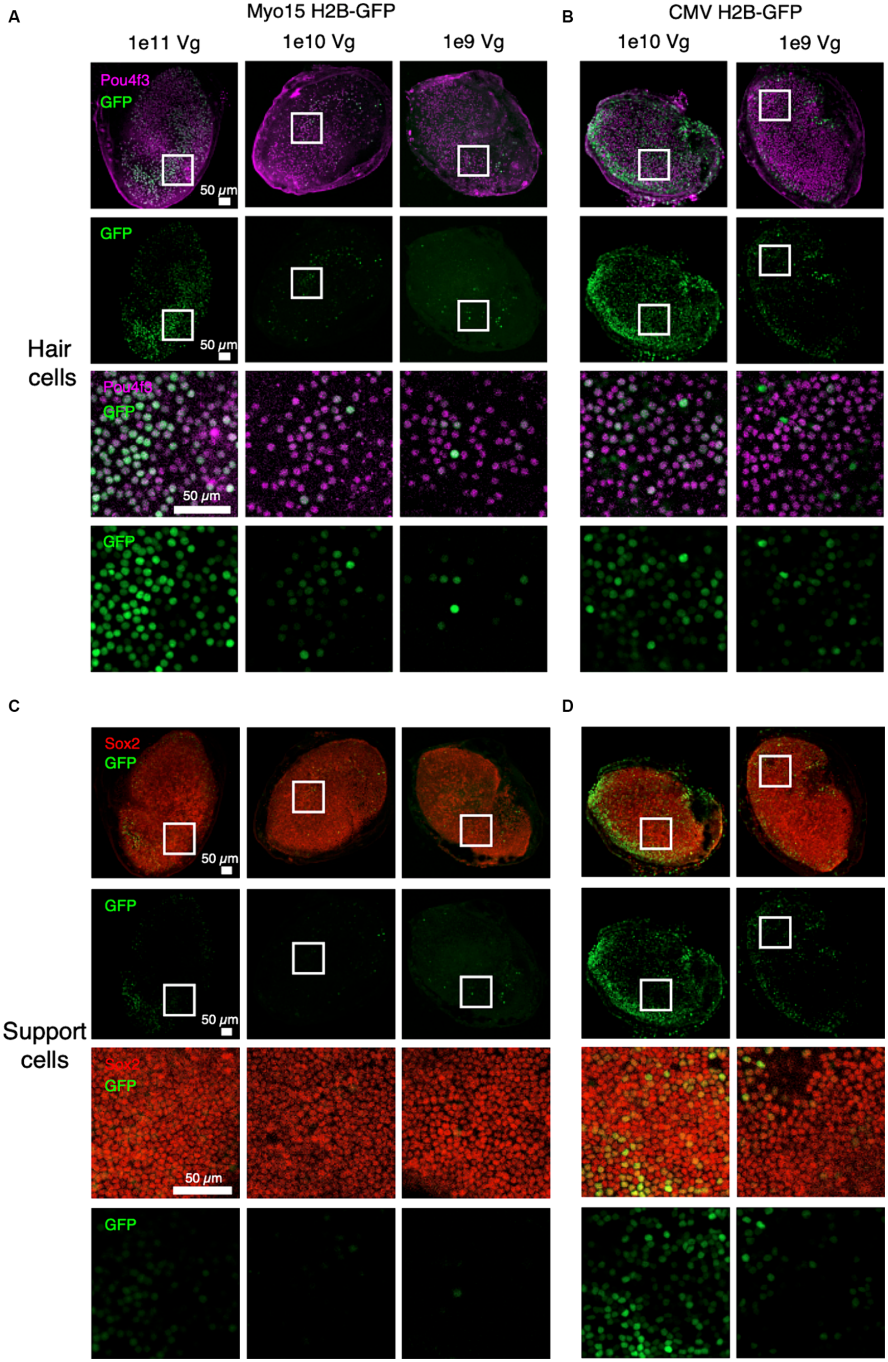
Specific expression of GFP driven by the *Myo15* promoter in cultured adult mouse utricles. Utricles from adult mice (C57BL6/J; *n* = 5 utricles, 3 animals per dose group) were cultured with AAV encoding GFP driven by either *Myo15* promoter at 1e11, 1e10, and 1e9 vg total dose **(A,C)** or driven by CMV promoter at 1e10 or 1e9 vg total dose **(B,D)**. Driving expression with the *Myo15* promoter in culture results in specific expression in utricular hair cells (labeled by Pou4f3; **A**), but little expression utricular support cells (labeled by Sox2; **B**). Driving expression with the CMV promoter results in expression in many cell types, including both utricular hair cells **(B)** and support cells **(D)**. Magenta, Pou4f3; Red, Sox2; Green, GFP.

To determine temporal dynamics of *OTOF* expression we first established whether the expression of GFP driven by the *Myo15* promoter localizes to vestibular hair cells. To do so we established *ex vivo* utricle cultures excised from the adult mouse temporal bone (see Materials and Methods). The utricles were infected with a single AAV containing an H2B-GFP transgene driven by either a ubiquitous *CMV* promoter (1e9 or 1e10 vg total dose) or a *Myo15* promoter (1e9, 1e10, and 1e11 vg total dose) and cultured for an additional 14 days (Figure 1). Use of the *Myo15* promoter resulted in restriction of the nuclear GFP signal to the hair cells of the sensory epithelia (marked by Pou4f3, Figure 1A) and results in minimal expression of GFP in support cells of the sensory epithelia (marked by Sox2, Figure 1C). In contrast, the CMV promoter resulted in ubiquitous expression throughout the utricle in both hair cells (Figure 1B) and support cells (Figure 1D). Increasing total vg dose of GFP driven by the *Myo15* promoter also results in increased expression in sensory hair cells (Figure 1A), but resulted in minimal expression outside the sensory epithelia (Figure 1C). This is in contrast with the CMV promoter which also resulted in dose dependent expression of GFP in support cells (Figure 1D). Given the specificity of GFP driven by the *Myo15* promoter to utricular hair cells, we establish that the utricle is a viable system exploring expression driven by the *Myo15* promoter in subsequent studies.

**Figure 2:**
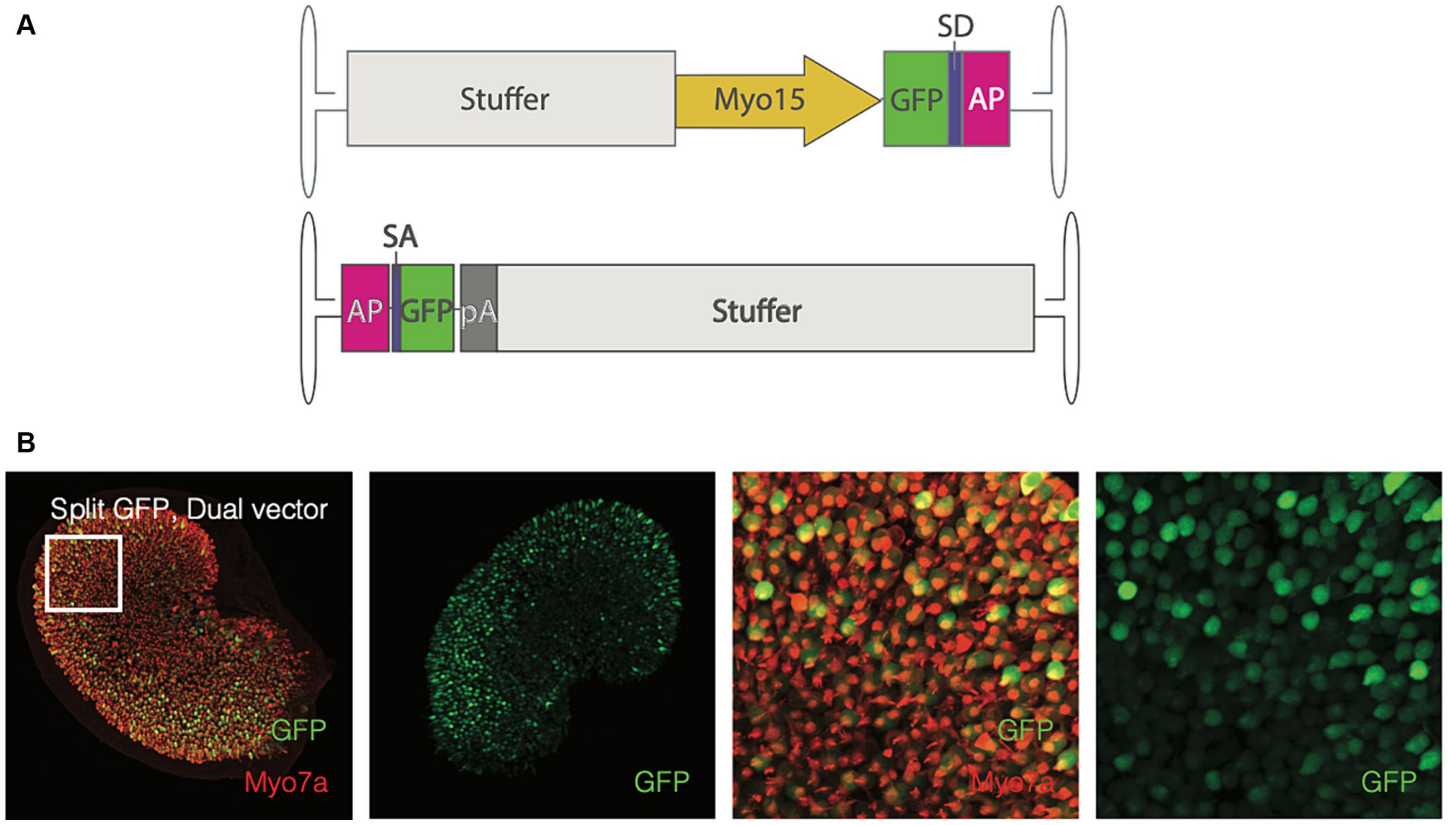
Adult utricles can be used to test dual AAVs. **(A)** Vector design of the split-GFP design (top, 5′ end; bottom, 3′ end). SD, splice donor; SA, splice acceptor; AP, alkaline phosphate recombinogenic region; pA, polyadenylation signal. Utricles from adult mice C57BL6/J; *n* = 5 utricles, 3 animals) were cultured with AAV encoding GFP driven by Myo15 promoter either in dual vector **(B)** at a total dose of 2e12 vg (1e12 per vector). Hair cells were labeled with Myo7a antibodies and the endogenous GFP signal is visualized. Columns 1–2: GFP expression in the whole sensory epithelia. Columns 3–4: A small region of the utricle (as indicated by square in the first column) to show the GFP expression in the hair cells. Red, Myo7a; Green, GFP.

Before exploring whether reconstitution of *OTOF* can be observed *ex vivo*, we sought to first evaluate the utility of the utricle explant as an *ex vivo* assay for observing dual vector recombination using split GFP. To do so we transduced cultured utricles with 1e12 vg each of dual vectors of AAV1 encoding a split GFP transgene driven by the *Myo15* promoter with the AP recombinogenic splice donor/accepter paradigm (Figure 2A, see Materials and Methods). Expression of GFP after dual vector recombination was robust and localized specifically to utricular hair cells (Figure 2B). Given ability to detect split GFP driven by the *Myo15* promoter, we establish that the cultured utricle is a viable system for observing dual vector recombination kinetics in hair cells.

### *OTOF* RNA and protein can be detected in individual utricles

To determine the amount of vestibular tissue needed to resolve *OTOF* transcripts we cultured C57BL6/J mouse utricle tissue with either 5e11 or 2.5e12 vg total of a dual vector otoferlin (DB-OTO) for 14 days. We utilize C57BL6/J tissue for this optimization since we can distinguish human OTOF transcripts from mouse *Otof* transcripts with our hOTOF10 probe (see Materials and Methods). After culture, we collected lysates from either 1 utricle, a pool of 2 or 3 utricles, or a pool of 5 cristae (Supplementary Figure S1A) using the Arcturus Picopure kit (Applied Biosystems, KIT0204). Isolated total RNA was purified, and cDNA was generated from resulting samples (see Materials and Methods). To determine relative amounts of *OTOF* transcripts we performed reverse transcription quantitative polymerase chain reaction RT-qPCR utilizing an *OTOF* specific probe as well as a probe for *Sox2* as a tissue level control gene targeting both hair and support cells for normalization. The *Sox2* probe allows us to normalize against the amount of surviving sensory epithelia after culture on a per utricle basis. Results indicate that the *OTOF* specific probe does not detect mouse otoferlin (*Otof*) transcripts. Furthermore, *OTOF* qPCR cycle thresholds were 27 or lower in all treated groups, versus cycle thresholds above 42 for non-reverse transcriptase controls (see Supplementary Sheet S1), suggesting that single utricles are sufficient for RT-qPCR readouts.

In a parallel experiment we also explored expression of *OTOF* by immunohistochemistry (IHC) in utricles from *Otof^Q828X/Q828X^* mice (see Materials and Methods for details on mouse model generation) after 14 days in culture with or without 5e11 vg of DB-OTO (Supplementary Figure S1B). We observed that our anti-OTOF antibody (see Materials and Methods) detects endogenous OTOF protein in FVB/NJ mice, but not in *Otof^Q828X/Q828X^* mice, demonstrating that our *Otof^Q828X/Q828X^* mice lack functional OTOF. Exogenous OTOF in hair cells of treated utricles can thus be resolved from endogenous OTOF at a dose of 5e11 vg after 14 days in culture (Supplementary Figure S1B, right panels).

### Expression of *OTOF* RNA and protein is dose dependent in utricular hair cells

**Figure 3:**
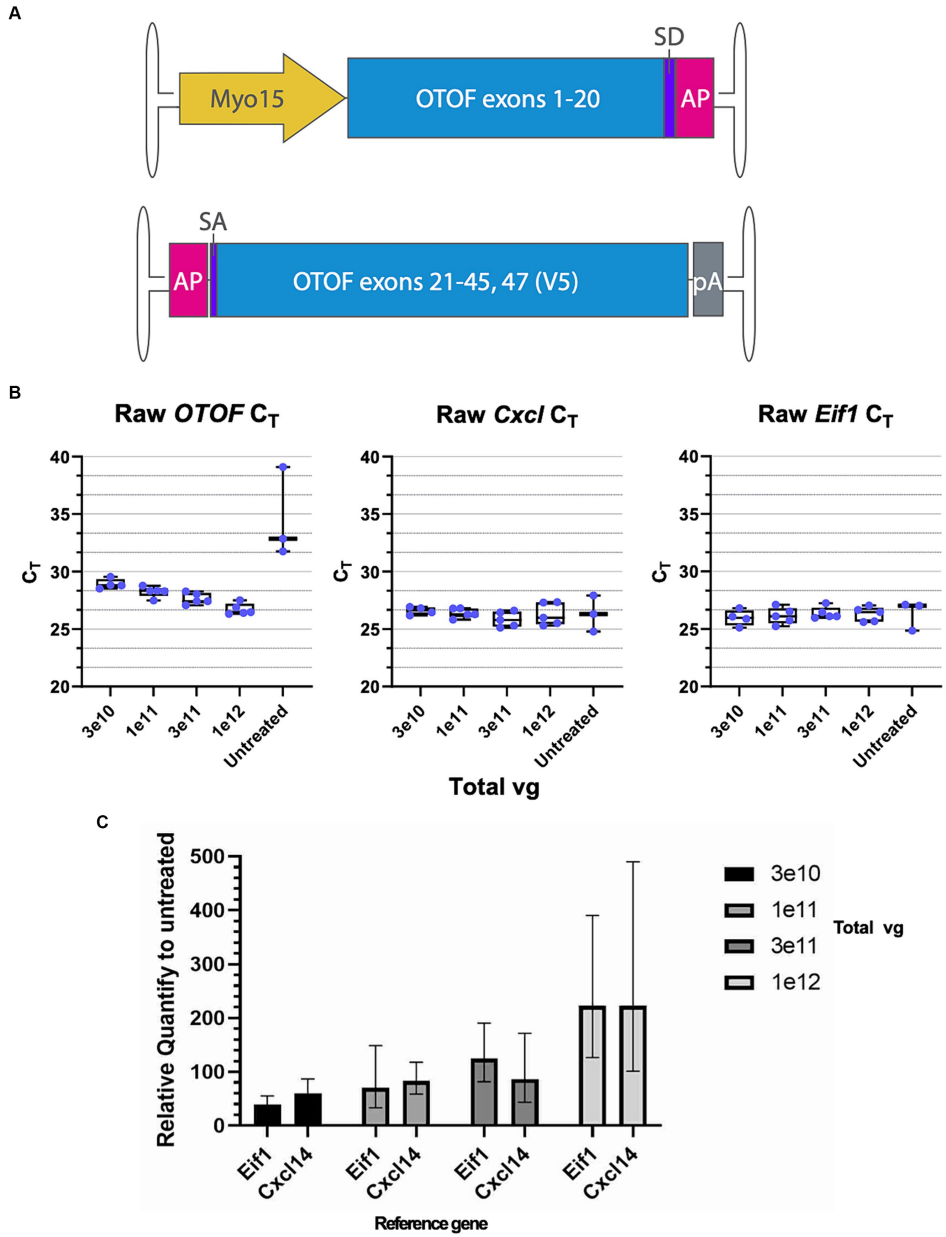
*OTOF* over-expression by AAV dose-dependently increases the *OTOF* RNA expression in the utricles. **(A)** Vector design of the dual vectors of DB-OTO. (top: 5′ vector; bottom: 3′ vector). **(B)** Real-time quantitative PCR was performed with cultured utricles with various doses of DB-OTO (C57BL6/J; *n* = 5 utricles, 3 animals per dose group) to quantify the *OTOF* RNA expression. Raw C_T_ values and box plots (median, quartiles, and range) for *OTOF* (left), *Cxcl14* (middle, hair cell reference gene) and *Eif1* (right, widely expressed reference gene) are plotted. Each dot represents one utricle and the average of two technical duplicates. **(C)** Relative quantify of *OTOF* expression in all 4 treated groups compared with untreated group. Error bars are calculated from 95% CI of ΔΔC_T_. See Supplementary Tables S1 and S2 for more details and statistics.

To further characterize DB-OTO (Figure 3A) in *ex vivo* tissue we cultured adult utricle tissue from *Otof^Q828X/Q828X^* mice at several total doses (3e10, 1e11, 3e11, and 1e12 vg) and performed either immunohistochemistry or RT-qPCR on individual utricles (see Materials and Methods) after 14 days in culture. We detected *OTOF* RNA at all doses and decreasing C_T_ values with increasing dose (Figure 3B; Supplementary Sheet S3). To account for tissue level differences on an individual utricle basis we utilized two tissue control genes, *Cxcl14* and *Eif1*, to normalize mRNA expression (Figure 3C middle and right panels; Supplementary Table S1: Δ C_T_ to two reference genes). *Eif1* was chosen to reflect the whole tissue condition (as it is expressed ubiquitously), and *Cxcl14* is specific to hair cells (Janesick et al., 2021) and thus these control markers allowed us to account for any tissue-to-tissue variation in the amount of surviving sensory epithelia. We anticipated *Cxcl14* to be a better control for comparing *OTOF* between tissues since its expression is restricted to hair cells by the *Myo15* promoter. After normalization by C_T_ for a reference gene (Supplementary Figure S2) and compared to untreated utricles (see Materials and Methods for Relative Quantity calculation), we observed a clear dose response correlating increasing *OTOF* RNA with increasing doses of DB-OTO (Figure 3C; Supplementary Table S2: ΔΔC_T_ to untreated group).

**Figure 4:**
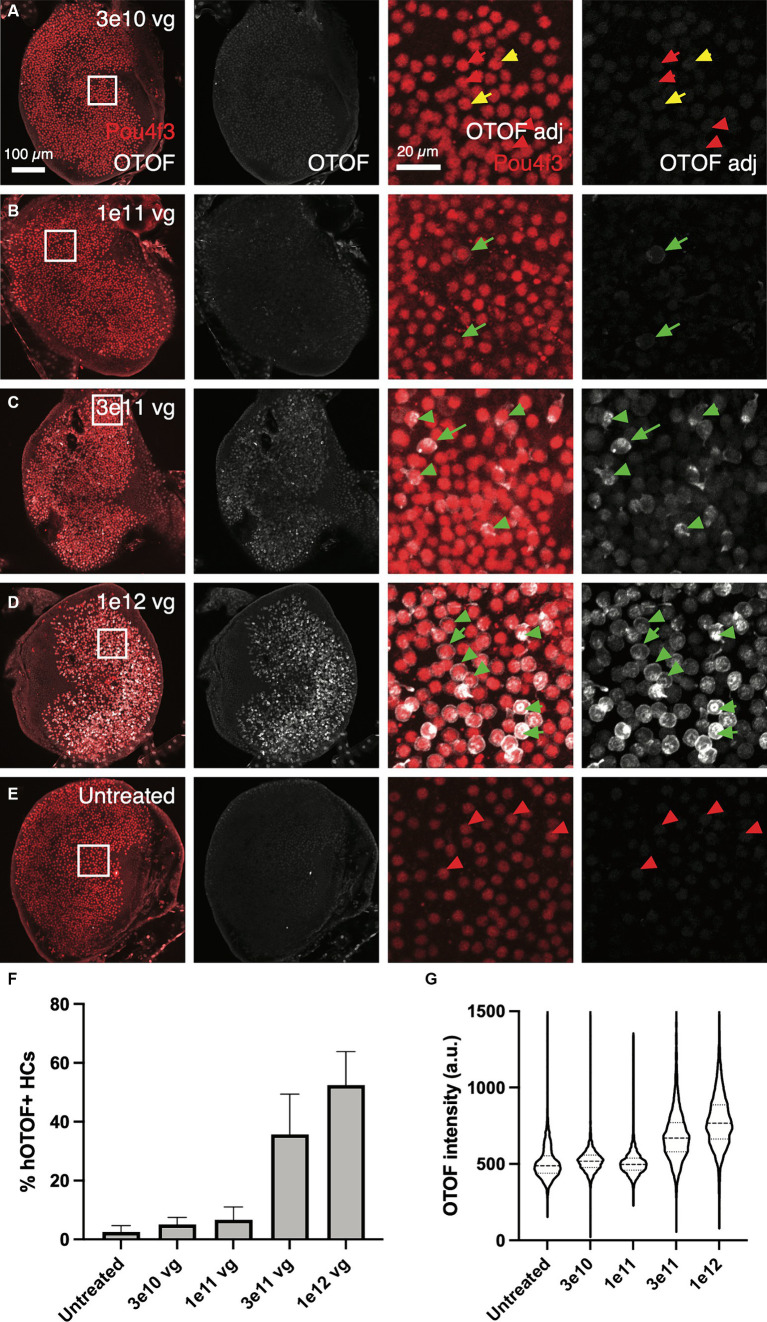
OTOF protein expression dose-dependently increased with AAV dose. AAVs encoding the 5′ and 3′ of the *OTOF* gene were delivered to the adult utricles from *Otof*^Q828X/Q828X^ mice (*n* = 4–5 utricles, 3 animals per dose group) **(A–D)** and cultured for 14 days at 1:1 ratio with various doses of DB-OTO. The OTOF protein was labeled with antibodies. Some utricles were cultured untreated as negative IHC control (E). First panel, OTOF protein signal overlaid with hair cells in utricles. Second panel, OTOF protein signal at various doses. OTOF was imaged with the same acquisition settings. Panels 3–4, zoom in view of OTOF expression in a small region of the utricle as shown in the square in first panel. Otof display is adjusted to show lack of signal in low-dose groups. Green arrow, OTOF-positive hair cells; red arrow, OTOF-negative hair cells; yellow arrow, possible OTOF-positive hair cells but with weak OTOF signals. (F,G) Quantification of the OTOF protein signal in hair cells. (F) The percentage of hair cells that are OTOF+ (mean ± S.D.). (G) Violin plots depicting the median (thick dashed lines) and quartiles (thin dashed lines) of OTOF intensity in absolute units (a.u., range 0–4,095 from 12-bit images) in OTOF-positive hair cells.

In addition to *OTOF* RNA, we also explored OTOF protein in utricles from *Otof^Q828X/Q828X^* mice. We observed increasing levels of OTOF in hair cells (labeled by POU4F3 positive nuclei) with increasing dose of DB-OTO (Figures 4A,D) and no OTOF observed in untreated controls (Figure 4E). To quantify this dose response, we determined the number of hair cells positive for OTOF after treatment and utilized Imaris to identify POU4F3-positive cells that were OTOF-positive (see Materials and Methods). We observed an increasing percentage of hair cells containing OTOF protein with increasing doses of DB-OTO (Figure 4F) as well as increasing intensity of OTOF signal in OTOF-positive hair cells with increasing dose (Figure 4G): 5.13 ± 2.36%, 6.71 ± 4.30%, 35.71 ± 13.69%, and 52.41 ± 11.45% at 3e10, 1e11, 3e11, and 1e12 vg total dose, respectively. Increasing doses of DB-OTO also had no significant effects on the survival of vestibular hair cells, suggesting little toxicity associated with hair cell transduction by AAV1 (Table 1).

**Table 1:** Number of hair cells and number of OTOF+ HCs (mean ± S.D.) by Pou4f3 labeling per dose of DB-OTO.

Dose condition	Number of hair cells (±S.D.)	Number OTOF+ HCs
3.00E+10 (*n* = 4)	2,324 ± 628	108 ± 26
1.00E+11 (*n* = 4)	2,390 ± 522	163 ± 123
3.00E+11 (*n* = 5)	2,203 ± 594	801 ± 343
1.00E+12 (*n* = 5)	2,374 ± 580	1,238 ± 394
Untreated (*n* = 4)	2,400 ± 973	59 ± 52

### Expression of recombined OTOF can be detected 3 days post application and plateaus by 14 days

**Figure 5:**
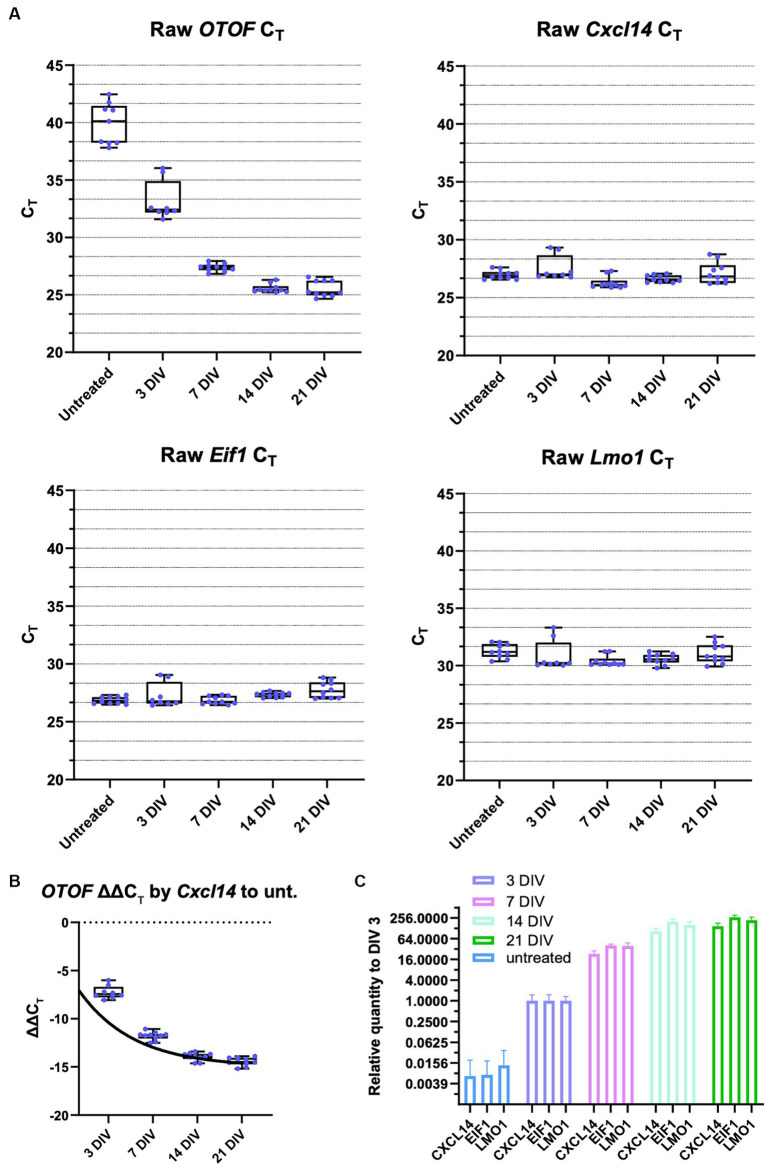
*OTOF* expression increases as the culture time and AAV expression time increases. RT-qPCR was performed using individual utricles (C57BL6/J; *n* = 5 biological replicates (utricles from 3 animals), two RT-qPCR technical replicates) treated with 5e11 vg of DB-OTO and cultured for 3, 7, 14, or 21 days. Each dot represents one technical replicate. **(A)** Raw CT values and box plots (median, quartiles, and range) for various durations are plotted. Cxcl14 and Lmo1 are hair cell markers, Eif1 is a widely expressed gene. **(B)** *OTOF* ΔΔC_T_. to untreated group normalized by *Cxcl14* and fit to an exponential plateau function. **(C)** Relative expression quantify at various days compared with untreated utricles.

After establishing dose response characteristics of DB-OTO in mouse utricular hair cells, we determined the length of time to detect full-length spliced *OTOF* mRNA transcripts and the time to reach plateau. To do this we cultured adult utricles from *Otof^Q828X/Q828X^* mice and isolated RNA (see Materials and Methods) at several time points after application of virus: 3 days, 7 days, 14 days, and 21 days. As a control, we additionally cultured 5 *Otof^Q828X/Q828X^* utricles and isolated RNA after 21 days in culture. Isolated RNA was purified, cDNA was generated from resulting samples (see Materials and Methods) and RT-qPCR was performed for *OTOF* as well as three control genes (*Eif1*, *Lmo1*, and *Cxcl14*) to account for variability between tissues (Figure 5A). These tissue control genes were utilized to normalize against tissue variability–– *Eif1* is ubiquitous and allows for normalization across the entire tissue while both *Cxcl14* and *Lmo1* (Deng et al., 2006) are hair cell specific and allowed us to normalize against sensory epithelia level variability. Utilizing two hair cell control genes provides additional confidence in the sample-to-sample hair cell survival and transduction variability. After normalizing by tissue control genes (Supplementary Figure S3) we observed high quantities of *OTOF* RNA at 7, 14, and 21 days post treatment compared to 3 days post treatment (Table 2: ΔC_T_ value to 3 reference genes). Statistical analysis confirmed significant increases in RNA levels with increasing time from initial dosing (see Tables 2, 3). *Post hoc* comparisons showed significant differences comparing the 3-day time point relative to the 7, 14, and 21 day time points for all reference genes tested. By 14 days in culture, *OTOF* RNA began to plateau with little significant change compared to 21 days in culture (Figures 5B,C; Table 3; Supplementary Sheet S3: ΔΔCt value to untreated group).

**Table 2:** qPCR results for time-course experiment, ΔC_T_ value to 3 reference genes.

Condition	ΔC_T_ ± standard deviation			ANOVA, multi-comparison *p* value^*^		
	Cxcl14	Eif1	Lmo1	Cxcl14	Eif1	Lmo1
Untreated	12.87 ± 1.871	13.01 ± 1.645	8.515 ± 1.769	N/A	N/A	N/A
3 DIV	5.613 ± 0.639	5.856 ± 0.655	2.277 ± 0.4577	<0.0001	<0.0001	<0.0001
7 DIV	1.087 ± 0.398	0.519 ± 0.169	−3.015 ± 0.398	<0.0001	<0.0001	<0.0001
14 DIV	−1.080 ± 0.384	−1.792 ± 0.295	−5.019 ± 0.470	<0.0001	<0.0001	<0.0001
21 DIV	−1.590 ± 0.406	−2.222 ± 0.237	−5.515 ± 0.364	<0.0001	<0.0001	<0.0001

^*^One-way ANOVA was performed confirming time in culture significantly affects the OTOF signal. Multiple comparisons (Dunnett’s test) were made by comparing each treated group to the untreated group.

**Table 3:** qPCR results for time-course experiment, ΔΔC_T_ value to 3 DIV group.

Condition	−ΔΔC_T_ ± standard deviation			ANOVA, multi-comparison *p* value^*^		
	Cxcl14	Eif1	Lmo1	Cxcl14	Eif1	Lmo1
3 DIV	−7.261 ± 0.6835	−7.155 ± 0.7006	−6.237 ± 0.4894	N/A	N/A	N/A
7 DIV	−11.79 ± 0.4198	−12.49 ± 0.1790	−11.53 ± 0.4198	<0.0001	<0.0001	<0.0001
14 DIV	−13.96 ± 0.4050	−14.80 ± 0.3110	−13.53 ± 0.4955	<0.0001	<0.0001	<0.0001
21 DIV	−14.47 ± 0.4288	−15.23 ± 0.2500	−14.03 ± 0.3846	<0.0001	<0.0001	<0.0001

^*^One-way ANOVA was performed confirming time in culture significantly affects the OTOF signal. Multiple comparisons (Dunnett’s test) were made by comparing each treated group to the 3 DIV group. Statistically, with all 3 markers, all groups are significantly different from each other, except for the pair DIV 14 and DIV 21 groups.

### Expression of OTOF protein plateaus by 14 days in *ex vivo* culture

**Figure 6:**
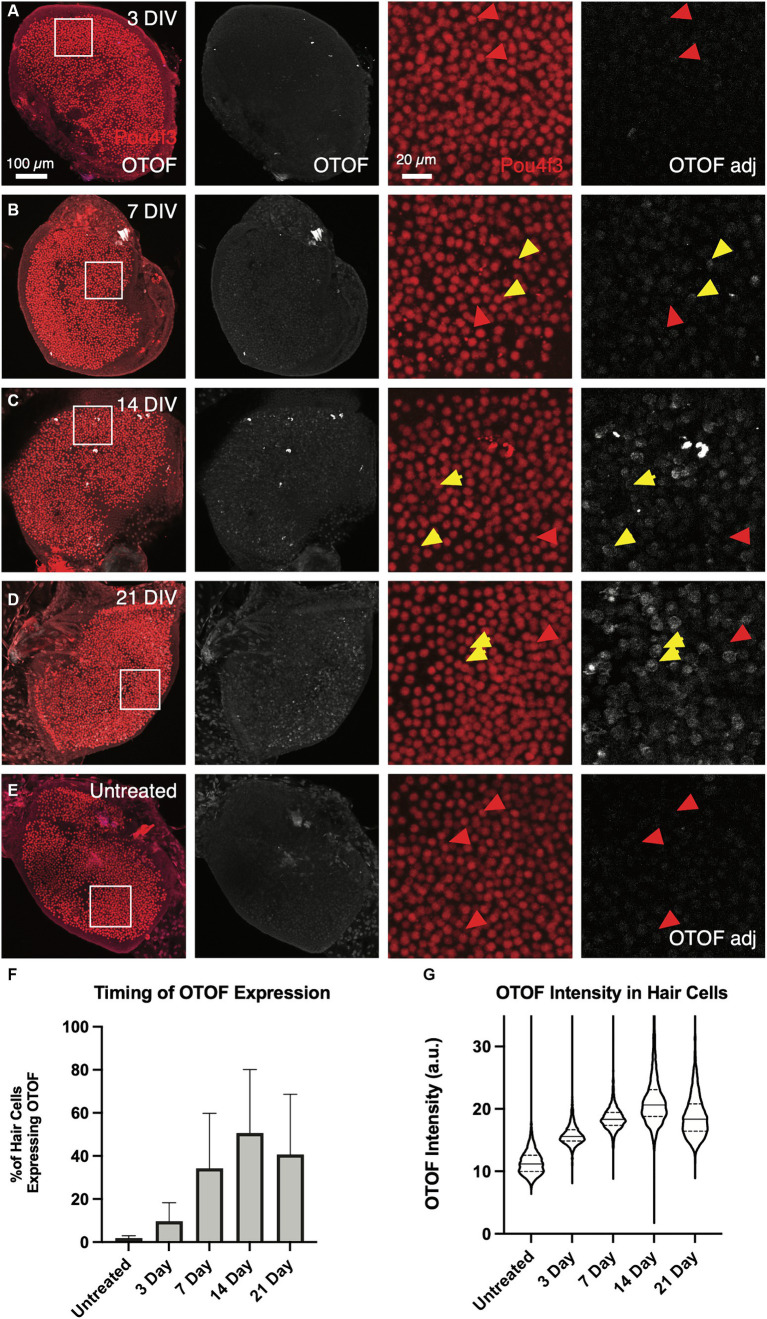
OTOF protein expression increases as the culture time increases. Adult utricles from *Otof*^Q828X/Q828X^ mice (*n* = 5 utricles, 3 animals per dose group) were treated with 5e11 vg DB-OTO and cultured for 3–21 days. The expression level of OTOF was analyzed **(A–D)**. As a negative control, utricles from Otof mutant mice were not treated but cultured for a total of 21 days (E) Columns 1–2: Overview of hair cells and OTOF expression in the whole utricle. Columns 3–4: A small region of the utricle (as shown in first column) is shown, highlighting the OTOF expression in the hair cells. Yellow arrow, OTOF+ hair cells; red arrow, HCs that are OTOF–. (F) The percentage of hair cells that are OTOF+ (mean ± S.D.). (G) Violin plots depicting the median (thick dashed lines) and quartiles (thin dashed lines) of OTOF intensity in absolute units (a.u., range 0–255 from 8-bit images) in OTOF-positive hair cells.

In addition to the time course of *OTOF* RNA, we explored OTOF protein in *Otof^Q828X/Q828X^* hair cells transduced with 5e11 vg of DB-OTO (2.5e11 DB-OTO 3′ and 2.5e11 DB-OTO 5′) over time (Figure 6). This dose of DB-OTO was chosen as it sits near the dose plateau presented in Figure 4. Adult utricles from *Otof^Q828X/Q828X^* and wildtype FVB/NJ mice were cultured for 21 days and utricles were fixed at several time points with and without application of virus at 3 days, 7 days, 14 days, and 21 days, then labeled for either POU4F3 or OTOF, and imaged by confocal microscopy (example utricles from each time point present in Figure 6; see Materials and Methods). After imaging, we quantified OTOF signal from each utricle using Imaris (see Materials and Methods). By 3 days post application of DB-OTO we observed few hair cells expressing OTOF protein (Figure 6A; 9.7 ± 8.6%, *n* = 4). By 7 days post application we observed significantly more hair cells expressing OTOF protein (Figure 6B; 34.3 ± 25.5%, *n* = 5). Between 14 and 21 days after application of DB-OTO to cultured utricles we began to see OTOF protein plateau in hair cells (Figures 6C,D; 50.6 ± 29.6%, *n* = 5 at 14 days, 40.7 ± 27.9% at 21 days, *n* = 4). In addition to the percentage of positive hair cells plateauing by 14 days post application of DB-OTO, we also quantified the intensity of the OTOF signal hair cells as a semi-quantitative readout of OTOF protein in hair cells. Figure 6G describes the intensity of all hair cells pooled from all utricles per time group. Compared to untreated utricles, treated utricles have hair cells with significantly greater OTOF intensity in treated groups, that increased with duration in culture and plateaued by 14 to 21 days post-treatment with DB-OTO.

### Small ratio deviations from 1:1 3′ to 5′ vector ratios have little impact on full length OTOF transcripts

**Figure 7:**
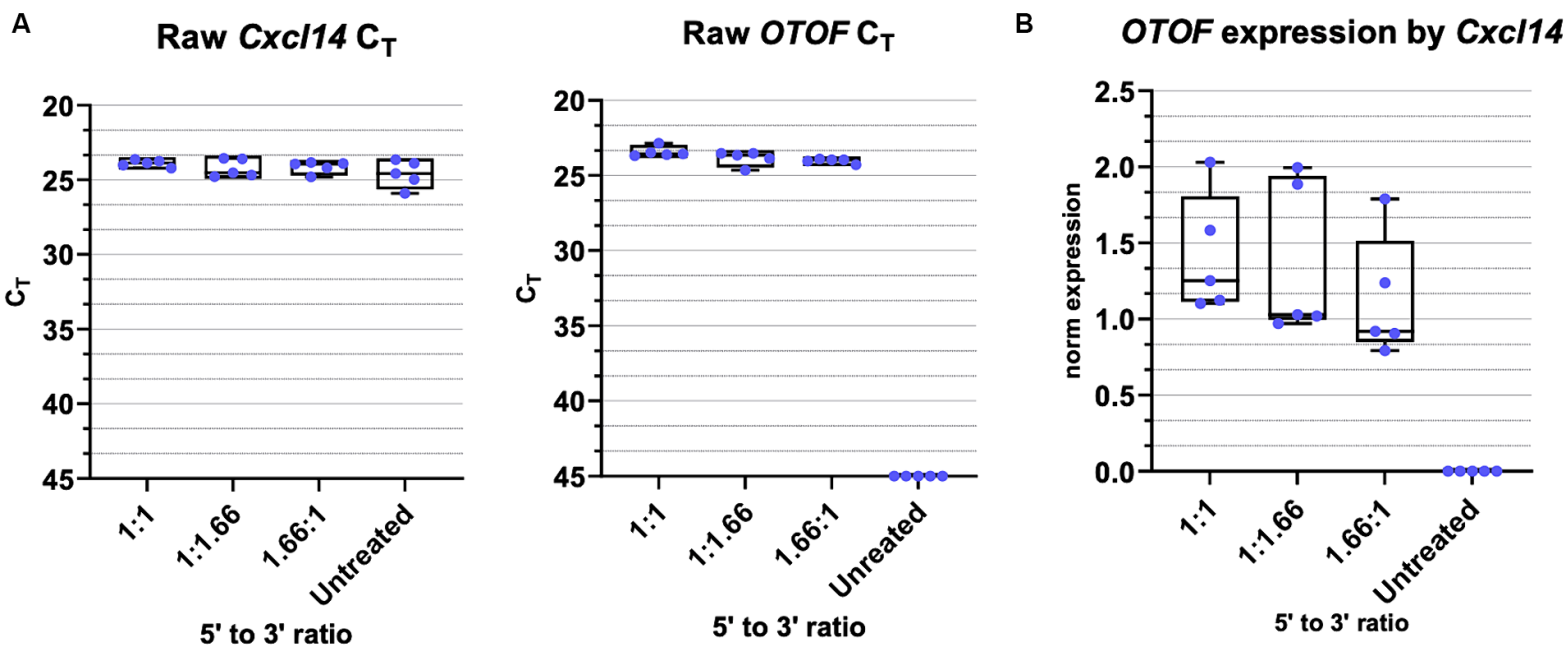
OTOF full length RNA with varied 5′ and 3′ vector ratios. **(A)** Real-time quantitative PCR was performed with cultured utricles (C57BL6/J; *n* = 5 biological replicates (utricles from 3 animals)) to quantify the *OTOF* RNA expression with different ratios of the 5′ and 3’ DB-OTO vectors. All dosing groups were cultured with the same 5e11 total vgs of DB-OTO. Raw C_T_ values and box plots (median, quartiles, and range) for *OTOF* (left) and *Cxcl14* (middle, hair cell reference gene) are plotted. Each dot represents one utricle and the average of two technical duplicates. **(B)** Full length *OTOF* expression normalized by hair cell control gene *Cxcl14* in all treated groups.

To evaluate the how differences in the dose of the 5′ and 3′ vectors might impact the amount of full-length *OTOF* RNA, we administered different ratios of the 5′ and 3′ vectors to excised mouse utricles from FVB/NJ mice, cultured for 14 days and evaluated *OTOF* transcript levels in these cultured utricles. All dosing groups were cultured with the same 5e11 vg total of DB-OTO and we explore ratios of 125% 5′ and 75% 3′ (and vice versa) to reflect possible error due to ddPCR titer measurement. After culture for 14 days, we lysed utricles, extracted and purified RNA, and performed RT-qPCR for *OTOF* (the full-length, human reconstituted *OTOF* transgene) as well as the hair cell specific control *Cxcl14* to account for tissue level variability. Supplementary Table S3, Supplementary Sheet S4, and Figure 7A includes the raw mean C_T_ values for each of the 5′:3′ vector ratios tested as well as the normalization of expression by *Cxcl14* (including ANOVA analysis of the normalized expression). As normalized by our hair cell control gene, *Cxcl14*, there was no significant difference between 5′:3′ ratio of 1.66:1.0, 1.0:1.66, and 1:1 (*p* > 0.05 for all groups versus 1:1) (Figure 7B). Thus, mixing error of up to +25% of one vector and − 25% of the other due to ddPCR accuracy has little significant effect on full length OTOF transcripts versus a 1:1 ratio.

### Expression of *OTOF* RNA plateaus with the same duration *in vivo*

**Figure 8:**
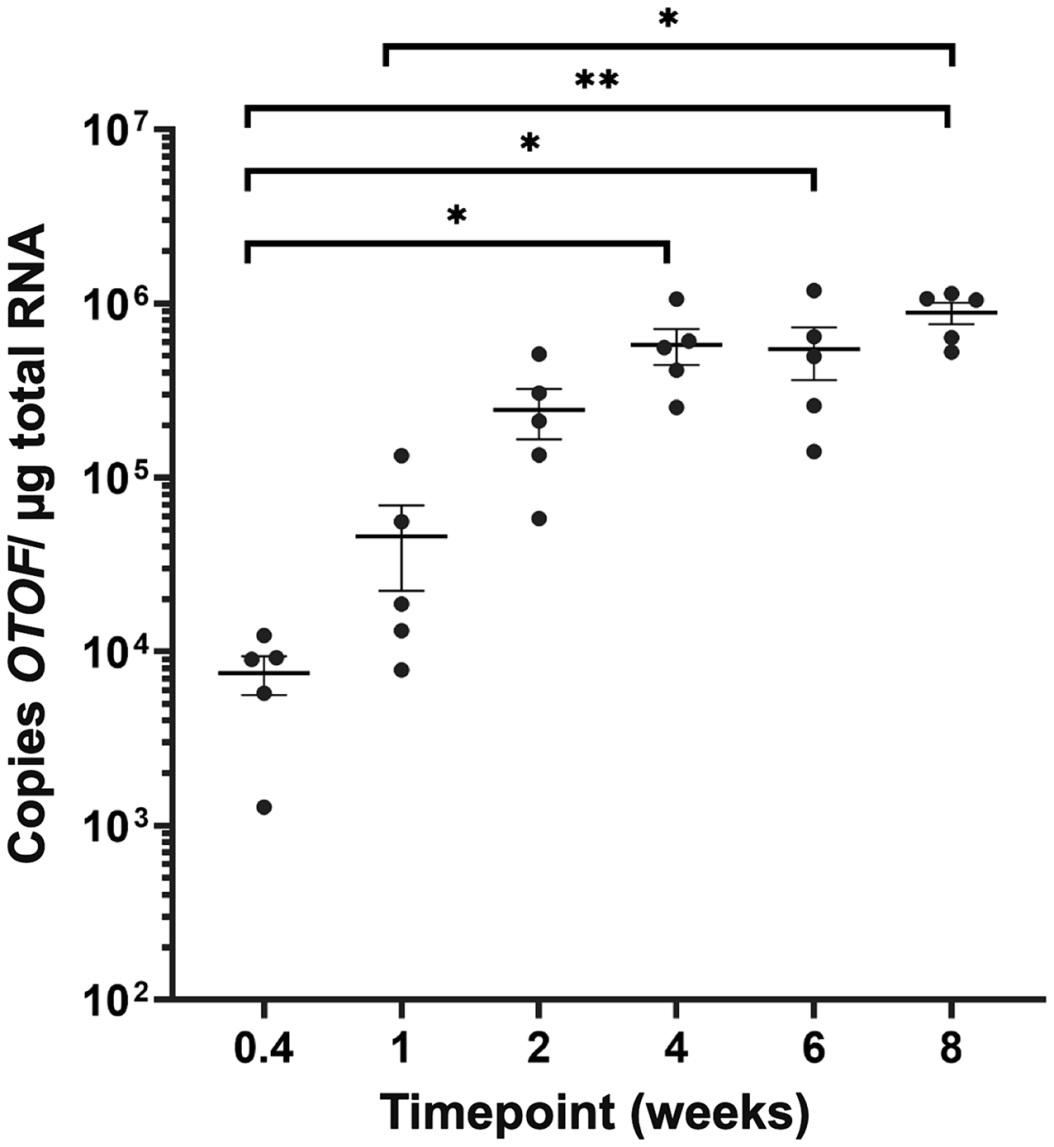
OTOF expression in the mouse cochlea *in vivo*. Quantification of *OTOF* expression in the mouse cochlea by RT-qPCR at different time points following DB-OTO administration *in vivo*. Absolute quantification of *OTOF* expression levels in the inner ear of adult mice (*n* = 5 mice/time point) following IC injection with 2E11 vg/ear of DB-OTO. Expression was quantified from 3 days to 8 weeks postinjection, and the results (±SEM) are expressed as *OTOF* copies/μg total RNA.

In a separate experiment, we administered DB-OTO to mice *in vivo* and explored the timing of plateau in copies of full length *OTOF*. *Otof^Q828X/Q828X^* mice were treated with a single unilateral intracochlear (IC) injection of DB-OTO at a dose level of 2E11vg/ear. Individual groups were sacrificed at 3 days, or 1, 2, 4, 6, or 8 weeks post-administration to evaluate *OTOF* transcript levels in the cochlea by RT-qPCR (see Materials and Methods*)*. We observed a time-dependent production of *OTOF* transcript *in vivo*. RT-qPCR results grouped by time point are shown in Figure 8, and the group mean, standard error of the mean (SEM), and percent coefficient of variation (%CV) are listed in Supplementary Table S4. Values for individual animals from each time point as well as mean C_T_ values are presented in Supplementary Sheet S5. The *OTOF* transgene was detectable starting at 3 days after injection, with a group mean of 7,525 copies/μg total RNA. Relative levels of transgene increased at the 1- and 2-week time points, reaching an average peak expression of 577,478 copies at 4 weeks post administration. Transgene levels remained elevated at 6 and 8 weeks, and no statistically significant differences were observed between the 4- through 8-week time points. Statistical analysis confirmed significant increases in RNA levels with increasing time from initial dosing (Kruskal–Wallis test, *p* = 2.80E-04). *Post hoc* comparisons showed significant differences in the 3-day time point relative to the 4 week time point (*p* = 0.026), the 3-day versus the 6-week time point (*p* = 0.033), the 3-day versus the 8-week time point (*p* = 0.0012), and the 1-week versus 8-week time point (0.018). Compared to our *ex vivo* RNA time course (Figure 5), *OTOF* levels plateaued in a similar time window *in vivo. Ex vivo* we observed a plateau between 2 and 3 weeks post treatment and *in vivo* we observed a plateau between 2 and 4 weeks post administration.

## Discussion

With the packaging limits of AAVs, several methods to rescue OTOF deficiency have been proposed. Protein splicing (Tang et al., 2023) and overstuffed AAV (Rankovic et al., 2021) were shown to rescue in OTOF in mouse models, but a high amount of partial product adds uncertainty for clinical applications. In contrast to overlapping approaches or transplicing approaches the addition of splice donor and acceptor sides flanking homologous regions, allow for and efficient recombination in the correct orientation and a high amount of full-length transcript (Ghosh et al., 2008; Dyka et al., 2014; Trapani et al., 2014; McClements and MacLaren, 2017; Reisinger, 2020; Rankovic et al., 2021). Therefore, the use of hybrid dual vectors as a gene therapy approach for delivering large genes *in vivo* provides a promising approach (Carvalho et al., 2017; Akil et al., 2019; Al-Moyed et al., 2019), while the timing, efficiency of transduction, generation of full-length mRNA, and protein expression with such methods have not previously been described.

To better characterize kinetics of expression driven by dual vectors we utilized a utricle-based culture method. With a post-mitotic sensory epithelia, the utricle provides a system to observe recombination events in originally infected cells. Using this method, we establish the utricle as viable system for demonstrating specificity of GFP expression driven by the *Myo15* versus *CMV* promoters (Figure 1). We demonstrated dose responsive specificity of GFP to vestibular hair cells using the *Myo15* promoter compared to the *CMV* promoter. Additionally, we demonstrated that dual-AAV1 vectors achieved efficient co-transduction in the mouse utricle with specific expression of GFP in vestibular hair cells, thus allowing us to observe the outcome of dual vector recombination events of split GFP (Figure 2A). Transduction with dual-AAV1 vectors was tolerable to mouse vestibular hair cells and resulted in robust expression when cultured *ex vivo* (Figure 2B), and minimal loss of hair cells when comparing 21 day culture in the untreated FVB utricles to those treated with DB-OTO (Table 1).

To achieve improvement in hearing threshold, a certain threshold of hair cells expressing the full, recombined transgene is likely necessary. For instance, otoferlin expression by dual-AAV6 vectors in ~30% of inner hair cells in *Otof*^−/−^ mice were shown to have hearing recovery (Al-Moyed et al., 2019). Achieving efficient transduction is likely limited by several factors—for instance, saturation of receptors of target receptors with viral capsids (Zengel and Carette, 2020) and other intracellular events (Domenger and Grimm, 2019; Zengel and Carette, 2020). While previous work has demonstrated efficient co-transduction of dual-AAVs in the utricle with high numbers of transduced hair and supporting cells after *in vivo* delivery (Chen et al., 2022), efficiency can be lower than with single vectors (Tan et al., 2019). Thus, we sought to explore the dose responsiveness of dual vectors delivering *OTOF* RNA (Figure 3) in the mouse utricle to achieve sufficient numbers of hair cells expressing OTOF protein (Figure 4) for functional recovery. Previous work in *Otof*^−/−^ mice (Al-Moyed et al., 2019) suggests transducing at least 30% of hair cells *in vivo* can drive hearing recovery. Our *ex vivo* system predicts that the local concentration needed to transduce at least ~30% of hair cells is 1.2e9 vg/μL (3e11 vg total dose in 250 μL media). The dose of 1.2e9vg/μL needed to achieve a sufficient number of hair cells *ex vivo* to drive recovery aligns with the 1.2e10 vg/μL dose needed to achieve recovery *in vivo* (Al-Moyed et al., 2019) given that the initial *in vivo* injected viral concentration would be significantly diluted from the injection point to the hair cell surface.

Between 1e11 vg and 3e11 vg total delivered vector to utricular hair cells we observed large increase in hair cells expressing OTOF protein (~28.3% difference in mean HCs expressing OTOF)—a significant increase compared to 3e10 vs. 1e11 vg (~1.5% difference in mean HCs expressing OTOF) delivered. The large difference between these dose groups suggests a minimal dose to achieve effective dual vector recombination in the *ex vivo* setting. The increase in total OTOF positive hair cells was also significantly larger between the 1e11 and 3e11 dose groups compared to 3e11 vg versus 1e12 vg total delivered (16.7% difference in mean HCs expressing OTOF), suggesting that by 1e12 vg delivered total to the culture we begin to encounter a dose plateau. There are several possibilities that may cause a plateau in the number of cells transduced to be less than total for the highest dose (Figures 4F,G). First, there is a chance that not all HCs will receive both vectors. Second, we know from previous work that HCs need to be overloaded with both vectors to achieve efficient recombination (Trapani et al., 2014) and there is a chance that any given cell did not receive enough of both vectors to achieve recombination and expression of the full length OTOF product.

Having demonstrated promoter specificity and dual vector recombination, we sought to determine the timing of generation of full-length *OTOF* mRNA in utricular hair cells. Previous work exploring timing of co-transduction dual-AAV delivery *in vivo* suggests that GFP protein can be observed at 2 weeks and maintained for up to 3 months in the adult mouse utricle by IHC (Chen et al., 2022), suggesting that evidence of full-length *OTOF* RNA should be detectible in less time. Here, we observed that full-length *OTOF* RNA can be detected in as little as 3 days post-administration, but OTOF protein was not detectible after 3 days in treated *Otof^Q828X/Q828X^* utricles (Figures 5, 6). While expression of OTOF protein was not detectable shortly after administration, it was detectable by 7 days post-administration. Thus, there is a lag between being able to detect OTOF protein even when *OTOF* mRNA is detectible after recombination. Between 14 to 21 days, we observed little significant difference in quantity of *OTOF* RNA in *Otof^Q828X/Q828X^* treated mouse utricles (Figure 5), suggesting a plateau in production of *OTOF* RNA. Similarly, we observed a plateau in the number of OTOF positive hair cells in treated utricles (Figure 6F) as well as the intensity of the OTOF signal as a semi-quantitative readout of OTOF protein (Figure 6G) between treated groups, suggesting the timing of protein quantity to plateau after dual-vector delivery using an *ex vivo* culture system. We further validated the utility of this *ex vivo* model system with an *in vivo* experiment that produced a similar plateau in mRNA expression (Figure 8). *In vivo* delivery to hair cells through the round window membrane is primarily in perilymph (Talaei et al., 2019) and thus different than delivery *ex vivo* where virus can enter hair cells both apically through the cuticular plate or basally. However, despite these delivery differences the timing of OTOF RNA is similar between the two systems. New delivery methods such as a cochleostomy or to the utricle to deliver to endolymph *in vivo* directly may be worth exploring in the future (Lee et al., 2020).

To better understand the relationship of the 5′ and 3′ vectors in producing full-length *OTOF* transcripts we also explored quantity of full-length *OTOF* RNA produced with different 5′ and 3′ ratios. At small deviations from a 1:1 vector ratio there was little effect on the quantity of *OTOF* produced (Figure 7; Supplementary Table S3). These results suggest that mixing error due to ddPCR accuracy has little significant effect on full length OTOF transcripts versus a 1:1 ratio.

In summary, we have established the mouse utricle as a viable system for exploring promoter specificity in hair cells, for observing the product of dual vector recombination events, characterizing the dose responsiveness and safety profile of delivery of OTOF to hair cells via dual vector AAV, and characterizing the kinetics of *OTOF* mRNA and protein in target cell populations. The utility of this model system is further validated by a similar mRNA expression plateau *in vivo*. Given that hair cells are post-mitotic, the utricle provides a framework to understand these dual vector recombination events in individual cells. In a proliferative, cell-culture based model, it would be difficult to observe these kinetics as genomes would be lost via cell division and may utilize different cellular mechanisms of recombination. Thus, understanding the properties of co-transduction with dual-AAV vectors in the utricle may enable a better estimate for the timing of recovery for other AAV based therapies in post-mitotic cell systems.

## Data availability statement

The original contributions presented in the study are included in the article/Supplementary material, further inquiries can be directed to the corresponding author. The sequences for DB-OTO and Myo15 promoter can be found in Patent Cooperation Treaty application WO2021087296.

## Ethics statement

The animal study was approved by Decibel Therapeutics Institutional Animal Care and Use Committee (IACUC). The study was conducted in accordance with the local legislation and institutional requirements.

## Author contributions

JS: Conceptualization, Data curation, Formal analysis, Investigation, Methodology, Supervision, Writing – original draft, Writing – review & editing. KS: Formal analysis, Investigation, Methodology, Writing – original draft, Writing – review & editing. AD'A: Formal analysis, Writing – review & editing. SC: Resources, Writing – review & editing. MD: Resources, Writing – review & editing. PS: Formal analysis, Writing – review & editing. NP: Conceptualization, Writing – review & editing. TG: Conceptualization, Writing – review & editing. TY: Formal analysis, Supervision, Writing – original draft, Writing – review & editing. JB: Conceptualization, Supervision, Writing – review & editing. AP: Conceptualization, Supervision, Writing – review & editing. LB: Conceptualization, Supervision, Writing – review & editing.
