# Supplementary material for: Recovery kinetics of dual AAV-mediated human otoferlin expression
Source: Front Mol Neurosci. 2024 Jun 17;17:1376128. doi: 10.3389/fnmol.2024.1376128 (PMC11215969; doi:10.3389/fnmol.2024.1376128)
Supplement: Supplementary file 1 [file Table_1.pdf]

**Recovery kinetics of dual AAV-mediated human Otoferlin expression**

Jonathan B. Sellon[1], Kathy S. So[1], Andrew D'Arcangelo[1], Sarah Cancelarich[2], Meghan C. Drummond[2], Peter G. Slade[1], Ning Pan[1], Tyler M. Gibson[1], Tian Yang[1], Joseph C. Burns[1], Adam T. Palermo[1], and Lars Becker[1]

[1]Decibel Therapeutics, Inc., Boston, MA 02215

[2]Regeneron Pharmaceuticals, Inc., Tarrytown, NY 10591

## Supplemental Tables

### Table S1: qPCR of dose experiment, delta $C_T$ value to 2 reference genes

| Condition | $\Delta C_T \pm$ standard deviation | | ANOVA, multi-comparison *p* value* | |
|---|---|---|---|---|
| | Cxcl14 | Eif1 | Cxcl14 | Eif1 |
| Untreated | 8.225 ± 2.855 | 8.234 ± 3.623 | N/A | N/A |
| 3E+10 | 2.325 ± 0.341 | 2.953 ± 0.318 | <0.0001 | 0.0004 |
| 1E+11 | 1.847 ± 0.409 | 2.099 ± 0.873 | <0.0001 | <0.0001 |
| 3E+11 | 1.793 ± 0.800 | 1.272 ± 0.494 | <0.0001 | <0.0001 |
| 1E+12 | 0.426 ± 0.916 | 0.436 ± 0.652 | <0.0001 | <0.0001 |

*: One-way ANOVA was performed and dose/treatment significantly affects the OTOF signal.

Multiple comparisons were made by comparing each treated groups to untreated group.

### Table S2: qPCR of dose experiment, delta delta $C_T$ value to untreated group

| Condition | $-\Delta\Delta C_T \pm$ standard deviation | | ANOVA, multi-comparison *p* value* | |
|---|---|---|---|---|
| | Cxcl14 | Eif1 | Cxcl14 | Eif1 |
| 3E+10 | -5.901 ± 0.341 | -5.282 ± 0.318 | N/A | N/A |
| 1E+11 | -6.379 ± 0.409 | -6.135 ± 0.873 | 0.5898 | 0.1459 |
| 3E+11 | -6.432 ± 0.799 | -6.962 ± 0.494 | 0.5144 | 0.0034 |
| 1E+12 | -7.800 ± 0.916 | -7.799 ± 0.652 | 0.0022 | <0.0001 |

*: One-way ANOVA was performed, and dose/treatment significantly affects the OTOF signal.

Multiple comparisons were made by comparing each treated group to 3e10.

**Table S3: qPCR results for time-course experiment, delta $C_T$ value to 3 reference genes**

| Condition | $\Delta C_T$ ± standard deviation | | | ANOVA, multi-comparison *p* value* | | |
|---|---|---|---|---|---|---|
| | Cxcl14 | Eif1 | Lmo1 | Cxcl14 | Eif1 | Lmo1 |
| Untreated | 12.87 ± 1.871 | 13.01 ± 1.645 | 8.515 ± 1.769 | N/A | N/A | N/A |
| 3 DIV | 5.613 ± 0.639 | 5.856 ± 0.655 | 2.277 ± 0.4577 | <0.0001 | <0.0001 | <0.0001 |
| 7 DIV | 1.087 ± 0.398 | 0.519 ± 0.169 | -3.015 ± 0.398 | <0.0001 | <0.0001 | <0.0001 |
| 14 DIV | -1.080 ± 0.384 | -1.792 ± 0.295 | -5.019 ± 0.470 | <0.0001 | <0.0001 | <0.0001 |
| 21 DIV | -1.590 ± 0.406 | -2.222 ± 0.237 | -5.515 ± 0.364 | <0.0001 | <0.0001 | <0.0001 |

*: One-way ANOVA (both Brown-Forsythe and Bartlett's test) was performed confirming time in culture significantly affects the OTOF signal. Multiple comparisons were made by comparing each treated group to the untreated group.

**Table S4: qPCR results for time-course experiment, delta delta $C_T$ value to untreated group**

| Condition | $-\Delta\Delta C_T$ ± standard deviation | | | ANOVA, multi-comparison *p* value* | | |
|---|---|---|---|---|---|---|
| | Cxcl14 | Eif1 | Lmo1 | Cxcl14 | Eif1 | Lmo1 |
| 3 DIV | -7.261 ± 0.6835 | -7.155 ± 0.7006 | -6.237 ± 0.4894 | N/A | N/A | N/A |
| 7 DIV | -11.79 ± 0.4198 | -12.49 ± 0.1790 | -11.53 ± 0.4198 | <0.0001 | <0.0001 | <0.0001 |
| 14 DIV | -13.96 ± 0.4050 | -14.80 ± 0.3110 | -13.53 ± 0.4955 | <0.0001 | <0.0001 | <0.0001 |
| 21 DIV | -14.47± 0.4288 | -15.23 ± 0.2500 | -14.03 ± 0.3846 | <0.0001 | <0.0001 | <0.0001 |

*: One-way ANOVA (both Brown-Forsythe and Bartlett's test) was performed confirming time in culture significantly affects the OTOF signal. Multiple comparisons were made by comparing each treated group to the 3 DIV group. Statistically, with all 3 markers, all groups are significantly different from each other, except for the pair DIV 14 and DIV 21 groups.

**Table S5: qPCR results for ratio variation**

| **Condition** | **Ratio DB-OTO 5' / DB OTO 3'** | **Mean of normalized expression** | **Lower 95%** | **Upper 95%** | **p-Value** |
|---|---|---|---|---|---|
| More 5' vector | 1.66:1.0 | 0.96 | 0.66 | 1.279 | 0.12 |
| Target | 1.0:1.0 | 1.41 | 1.14 | 1.692 | 1.0 |
| More 3' vector | 1.0:1.66 | 1.38 | 1.11 | 1.65 | 0.9997 |
| Control | Untreated | 6.13e-7 | -0.31 | 0.31 | <0.0001 |

**Table S6: Summary of Group Mean OTOF Copy Number in the Cochlea of *Otof*$^{Q828X/Q828X}$ Mice at Different Time Points Following DB-OTO Treatment**

| **Time point** | **Cochlea (N = 5/time point)** | | |
|---|---|---|---|
| | **Mean (copies/µg RNA)** | **SEM** | **%CV** |
| 3 days | 7524.52 | 1881.07 | 55.90 |
| 1 week | 45,802.74 | 23,484.78 | 114.65 |
| 2 weeks | 244,170.08 | 78,435.27 | 71.83 |
| 4 weeks | 577,477.82 | 135,191.04 | 52.35 |
| 6 weeks | 544,982.71 | 182,673.95 | 74.95 |
| 8 weeks | 882,307.31 | 125,502.71 | 31.81 |
