# Supplementary material for: Recovery kinetics of dual AAV-mediated human otoferlin expression
Source: Front Mol Neurosci. 2024 Jun 17;17:1376128. doi: 10.3389/fnmol.2024.1376128 (PMC11215969; doi:10.3389/fnmol.2024.1376128)
Supplement: Supplementary file 7 [file Image_1.PDF]

Recovery kinetics of dual AAV-mediated human Otoferlin expression

Jonathan B. Sellon[1], Kathy S. So[1], Andrew D'Arcangelo[1], Sarah Cancelarich[2], Meghan C. Drummond[2], Peter G. Slade[1], Ning Pan[1], Tyler M. Gibson[1], Tian Yang[1], Joseph C. Burns[1], Adam T. Palermo[1], and Lars Becker[1]

[1]Decibel Therapeutics, Inc., Boston, MA 02215

[2]Regeneron Pharmaceuticals, Inc., Tarrytown, NY 10591

Supplemental Information

Supplemental Figures

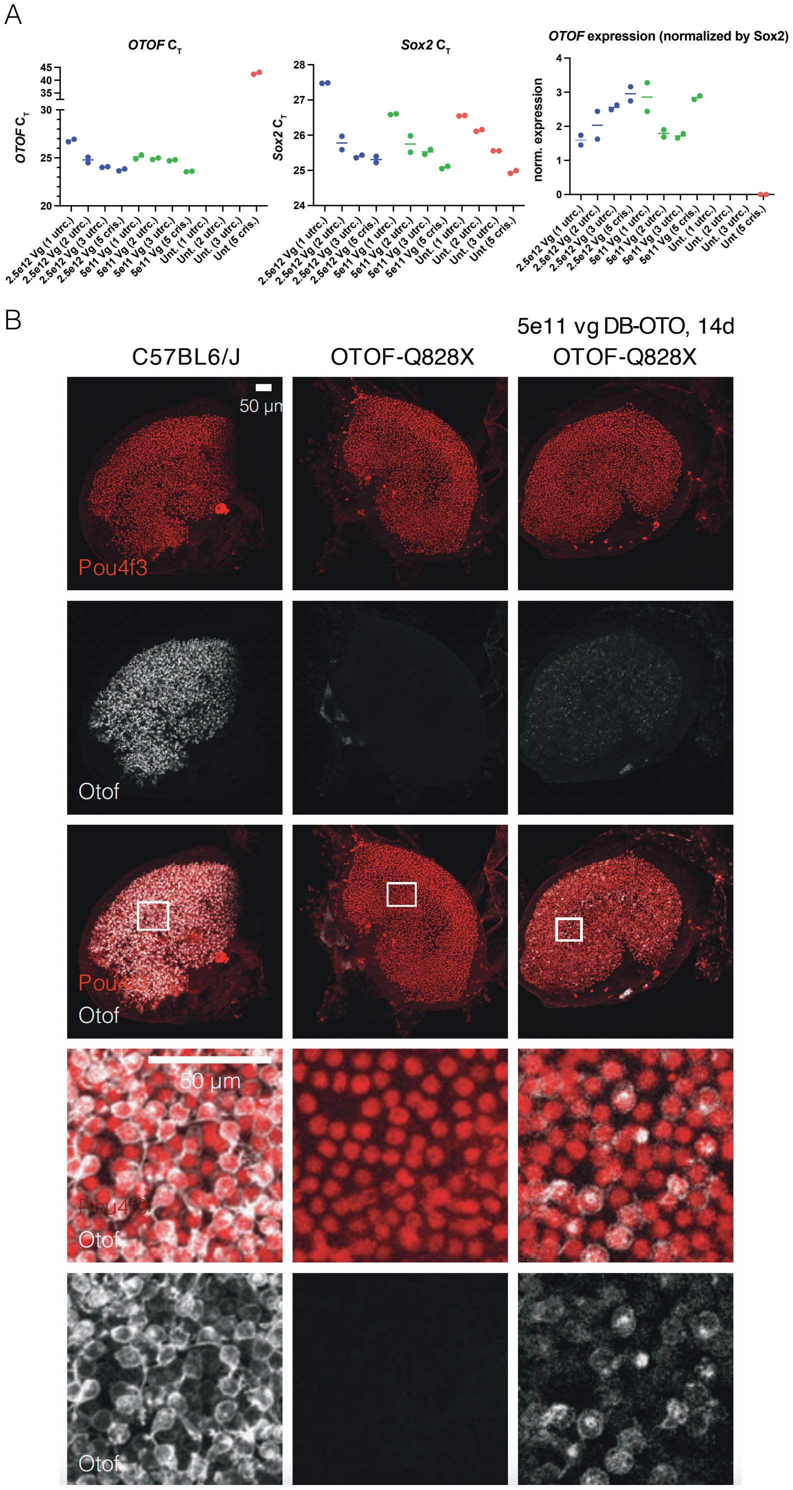

Figure S1: Establishing methods for detecting recombined, full-length OTOF RNA and protein in culture. (A) Real-time quantitative PCR was performed with utricles cultured with

AAV encoding the 3' and 5' ends of OTOF driven by Myo15 (at either 5e11 or 2.5e12 total vg) to determine feasibility and quantity of tissue needed to accurately read out recombined OTOF mRNA. C_T values for *OTOF* (left) and *Sox2* (center, hair cell reference gene) are plotted for 1, 2, or 3 utricles and 5 cristae pooled after culture. Normalized OTOF expression (right) normalizes OTOF expression based on the *Sox2* reference gene to account for tissue variability. (B) Utricles from C57BL6/J (left) and *Otof*$^{Q828X/Q828X}$ mice were cultured for 14 days with (right) and without (center) AAVs encoding the 5' and 3' of the *OTOF* gene. No OTOF expression was observed in cultured *Otof*$^{Q828X/Q828X}$ utricles without AAV (center), but significant expression was seen localized to hair cells (labeled by Pou4f3, right).

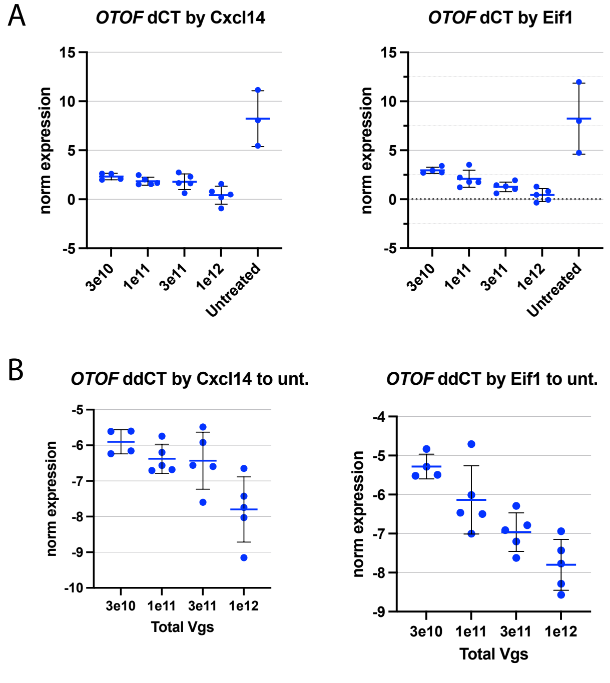

Figure S2: *OTOF* ΔC_T normalized and $\Delta\Delta C_T$ to untreated. (A) *OTOF* ΔC_T determined by either *Cxcl14* (left) or *Eif1* (right) reference genes at four doses (3e10, 1e11, 3e11, and 1e12 vgs) of DB-OTO or untreated. (B) *OTOF* $\Delta\Delta C_T$ to the untreated group determined by either *Cxcl14* (left) or *Eif1* (right) reference genes at four doses of DB-OTO.

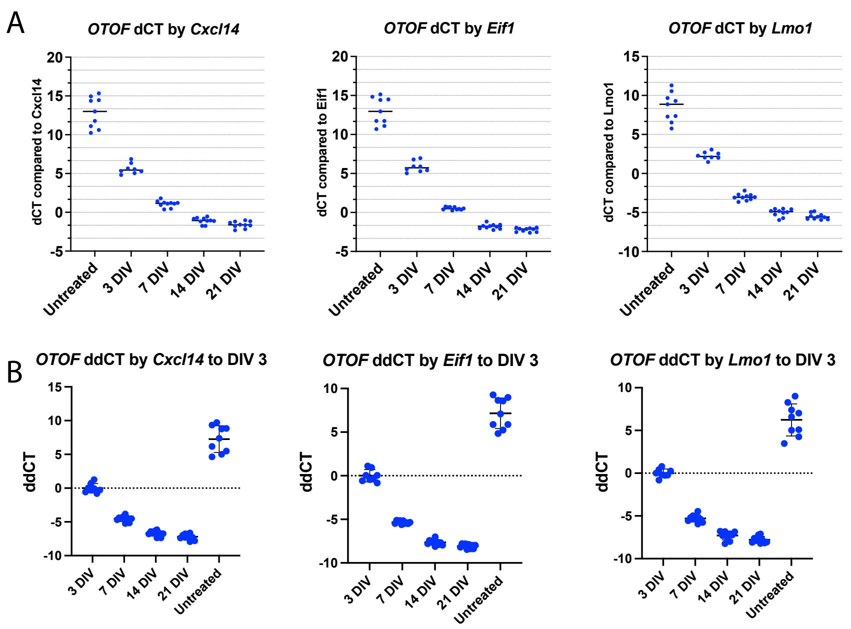

Figure S3: OTOF expression normalized by Eif1, Cxcl14, and Lmo1 and $\Delta\Delta C_T$ to 3 DIV.

(A) OTOF ΔC_T determined by either *Cxcl14* (left), *Eif1* (middle), or *Lmo1* (right) reference genes at four time points (3, 7, 14, and 21 days) post treatment with 5e11 vg DB-OTO or untreated. (B) OTOF $\Delta\Delta C_T$ to the untreated group determined by either *Cxcl14* (left), *Eif1* (middle), or *Lmo1* (right) reference genes at four time points (3, 7, 14, and 21 days) post treatment with DB-OTO or untreated.
